# Laminarin Induces Apoptosis of Human Colon Cancer LOVO Cells through a Mitochondrial Pathway

**DOI:** 10.3390/molecules17089947

**Published:** 2012-08-20

**Authors:** Yu Bin Ji, Chen Feng Ji, He Zhang

**Affiliations:** 1 Engineering Research Center of Natural Anticancer Drugs, Ministry of Education, Harbin University of Commerce, Harbin 150076, China; E-Mail: jyb@hrbcu.edu.cn; 2 Center of Research on Life Science and Environmental Science, Harbin University of Commerce, Harbin 150076, China; E-Mail: zhanghe84@163.com

**Keywords:** laminarin, human colon cancer, apoptosis, mitochondrial pathway

## Abstract

Many scientific studies have shown that laminarin has anti-tumor effects, but the anti-tumor mechanism was unclear. The purpose of this study was to investigate the effect of laminarin on the induction of apoptosis in human colon cancer LOVO cells and the molecular mechanism involved. LOVO cells were treated with different concentrations of laminarin at different times. Morphology observations were performed to determine the effects of laminarin on apoptosis of LOVO cells. Flow cytometry (FCM) was used to detect the level of intracellular reactive oxygen species (ROS) and pH. Laser scanning confocal microscope (LSCM) was used to analyze intracellular calcium ion concentration, mitochondrion permeability transition pore (MPTP) and mitochondrial membrane potential (MMP). Western blotd were performed to analyze the expressions of Cyt-C, Caspase-9 and -3. The results showed the apoptosis morphology, which showed cell protuberance, concentrated cytoplasm and apoptotic bodies, was obvious after 72 h treatment. Laminarin treatment for 24 h increased the intracellular level of ROS and Ca^2+^; decreased pH value; activated intracellular MPTP and decreased MMP in dose-dependent manners. It also induced the release of Cyt-C and the activation of Caspase-9 and -3. In conclusion, laminarin induces LOVO cell apoptosis through a mitochondrial pathway, suggesting that it could be a potent agent for cancer prevention and treatment.

## 1. Introduction

Colon cancer is a common malignant tumor in the digestive tract and one of the four most common malignant tumors throughout the World. With the rise in peoples’ living standards and the changes in their diet structure, the incidence of this cancer has been increasing year by year. At present, treatment for colon cancer mainly takes the form of surgical operation, supplemented by whole-body radiotherapy or chemotherapy, but the outcome of such treatment is far from ideal. In recent years, a consensus has developed internationally, as well as in China, that effective anti-tumor drugs should be sought in natural medicine.

**Figure 1:**
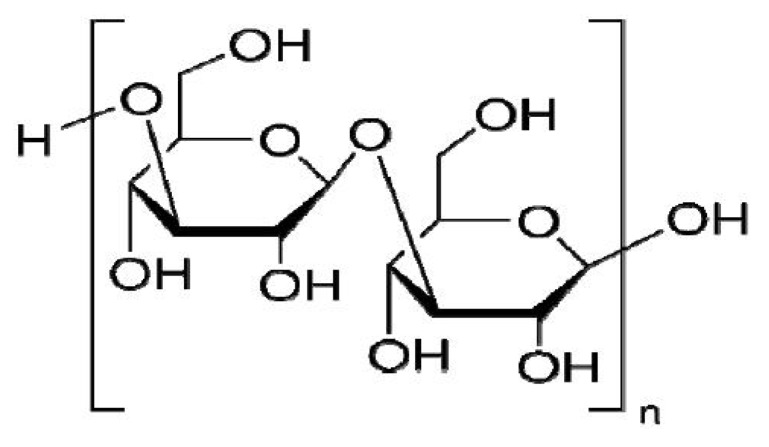
Structural formula of laminarin.

Many of the activities of polysaccharides, such as their anti-tumor and immunological regulation effects, are closely associated with cell apoptosis, but only some preliminary work has been done on the mechanism of their anti-tumor effect. Laminarin (Figure 1) is an active component extracted and isolated from the dry thallus of *Laminaria japonica* Aresch of the Laminariaceae family or *Ecklonica kurome* Okam. of the Alariaceae family [1]. It is made up of β (1→3)-glucan with β (1→6)-linkages. The anti-tumor effect of laminarin has been reported [2,3,4], and we have found laminarin could induce LOVO cells apoptosis [5], Papk *et al.* reported that laminarin could inhibit HT-29 cell growth by decreasing cell proliferation and induce apoptosis via death receptor pathway and IGF-IR pathway.

There are many cell apoptosis pathways, and many pathways have interactions. Based on our early work, in the present *in vitro* experiment, we further study whether laminarin can induce LOVO cells apoptosis through one of classic apoptosis pathways—the mitochondrial pathway—which has not been reported till now. Moreover, this experiment will broaden laminarin development and application.

## 2. Results and Discussion

### 2.1. Apoptosis Morphology Observation

**Figure 2:**
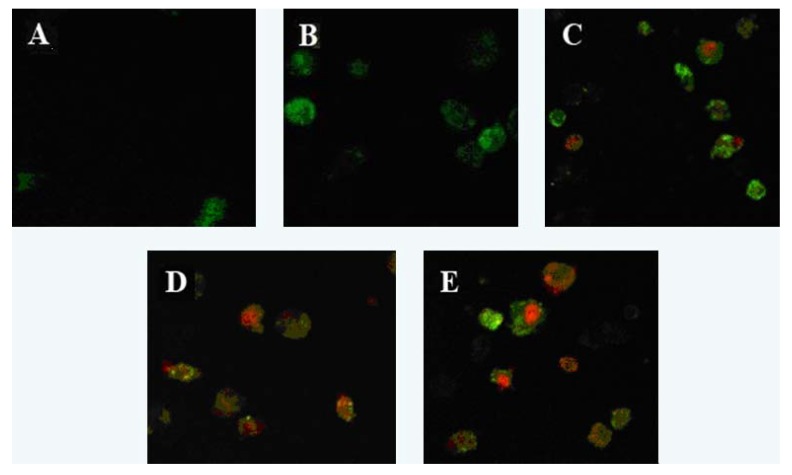
Effect of laminarin on LOVO cells morphology. (**A**) Cells were treated as control group; (**B**) Cells were treated with 400 µg·mL^−1^ laminarin; (**C**) Cells were treated with 800 µg·mL^−1^ laminarin; (**D**) Cells were treated with 1,600 µg·mL^−1^ laminarin; (**E**) Cells were treated with 5 µg·mL^−1^ HCPT.

Laser scanning confocal microscopy showed that cells in the control group were not dyed by Annexin V-FITC or PI, which showed fluorescence. After treatment with laminarin for 24 h, cells in the low concentration group were dyed by Annexin V-FITC and showed green fluorescence, which indicates the early phase of apoptosis. As laminarin concentration increased, cells in the middle and high groups were dyed by Annexin V-FITC and PI and showed red fluorescence inside with green fluorescence outside, which corresponds to the late phase of apoptosis (Figure 2).

### 2.2. Effect of Laminarin on Reactive Oxygen Species (ROS), Calcium Ion Concentration (Ca^2+^) and pH Value in LOVO Cells

#### 2.2.1. Effect of Laminarin on ROS in LOVO Cells

**Figure 3:**
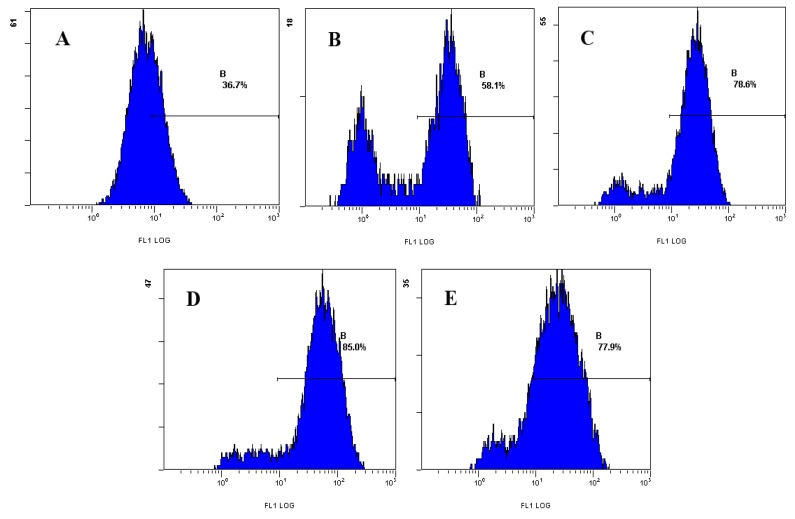
Effect of laminarin on ROS of LOVO cells by FCM analysis. (**A**) Cells were treated as control group; (**B**) Cells were treated with 400 µg·mL^−1^ laminarin; (**C**) Cells were treated with 800 µg·mL^−1^ laminarin; (**D**) Cells were treated with 1,600 µg·mL^−1^ laminarin; (**E**) Cells were treated with 5 µg·mL^−1^ HCPT.

Analysis with FCM showed that after treatment with different concentrations of laminarin for 24 h, the mean fluorescence intensity (MFI) of intracellular ROS increased significantly (*p* < 0.01), and with the increasing drug concentration, the level of ROS increased accordingly; the percentages were 58.1%, 78.6%, 85.1%, respectively, whereas in the control group it was 36.5% (Table 1 and Figure 3).

#### 2.2.2. Effect of Laminarin on Ca^2+^ in LOVO Cells

**Figure 4:**
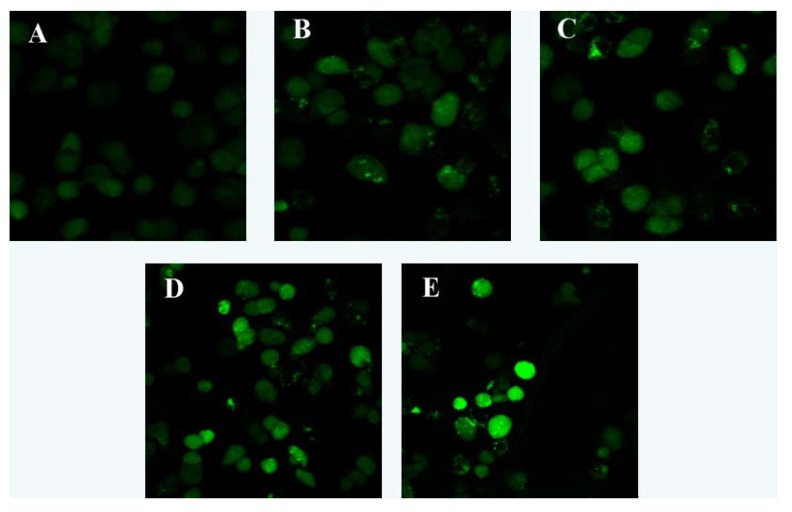
Effects of laminarin on intracellular fluorescent intensity of [Ca^2+^] in LOVO. (**A**) Cells were treated as control group; (**B**) Cells were treated with 400 µg·mL^−1^ laminarin; (**C**) Cells were treated with 800 µg·mL^−1^ laminarin; (**D**) Cells were treated with 1,600 µg·mL^−1^ laminarin; (**E**) Cells were treated with 5 µg·mL^−1^ HCPT.

The results showed that after treatment with different concentrations of laminarin for 24 h, the fluorescence intensity of intracellular Ca^2+^ increased significantly compared with control group (*p* < 0.01). With the increasing drug concentration, Ca^2+^ concentration increased accordingly (Table 2 and Figure 4).

#### 2.2.3. Effect of Laminarin on pH in LOVO Cells

**Figure 5:**
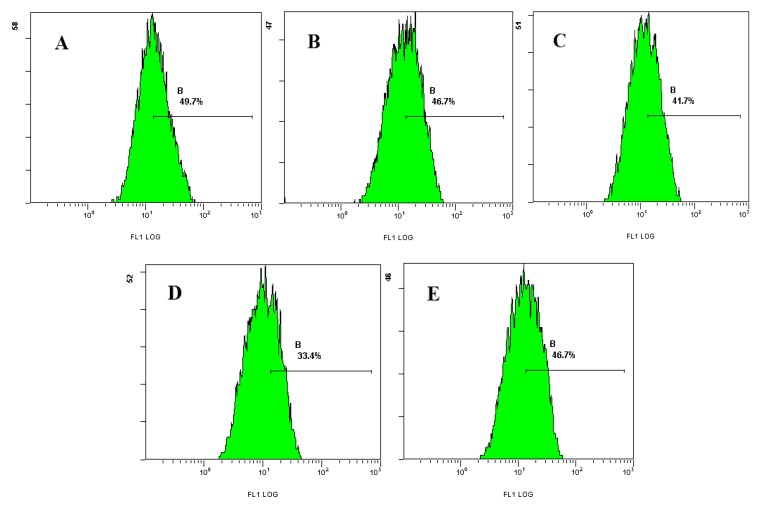
Effect of laminarin on pH of LOVO cells by FCM analysis. (**A**) Cells were treated as control group; (**B**) Cells were treated with 400 µg·mL^−1^ laminarin; (**C**) Cells were treated with 800 µg·mL^−1^ laminarin; (**D**) Cells were treated with 1,600 µg·mL^−1^ laminarin; (**E**) Cells were treated with 5 µg·mL^−1^ HCPT.

The results showed that after treatment with different concentrations of laminarin for 24 h, the mean fluorescence intensity of intracellular pH decreased significantly. With the increasing drug concentration, pH value decreased accordingly (46.7%, 41.7%, 33.4%, respectively, whereas in the control group it was 49.7%; Table 3 and Figure 5).

Imbalance of tumor cells’ apoptosis regulation is one of the main reasons for the occurrence of tumors. The mitochondrion, as the cell’s base for aerobic respiration and site of energy supply, plays an important role in the occurrence of cell apoptosis [6,7]. The mitochondrion contains certain materials closely associated with apoptosis, such as Ca^2+^, ROS, Cyt-C and AIF. Stimulated by signals, the permeability of the mitochondrion increases, leading to a crucial series of changes, including the lowering of mitochondrial membrane potential (ΔΨ_m_), changes in the oxidation-reduction state, interference by members of the Bcl-2 family, the release of Cyt-C, and the activation of Caspase [8,9]. The transmission of different signals is finally focused on the mitochondrion for activation or inhibition of these events, and plays to regulate apoptosis through relevant signal transmission pathways.

The experimental results show that the ROS level in LOVO cells rises after treated with laminarin. Increase in ROS can on the one hand damage mitochondrial membranes, leading to the opening of mitochondrial permeability transition pores (MPTP), releasing Ca^2+^ and Cyt-C, and on the other hand increase the expression of Bax and produce homodimers that act upon MPTP, resulting in the lowering of mitochondrion transmembrane potential and the release of apoptosis-promoting factors, thus inducing apoptosis [10,11]. Laminarin can also increase cytoplasm Ca^2+^, which further promotes the opening of MPTP, leading to a lowering of membrane potential. It can also activate a series of proteins related to apoptosis mediated by mitochondria, thus inducing apoptosis [12]. Furthermore, increase in Ca^2+^ can also lead to an increase in intracellular H^+^, resulting in acidification in the cells [13]. Experimental results also show that laminarin can induce acidification in the cell, which on the one hand can activate DNase II and DNA degradation, and on the other facilitate the release of Cyt-C from mitochondria and the automatic execution of the procedure for apoptosis by Caspase [14,15]. In the cells, ROS, Ca^2+^, and pH interact with one another, resulting in magnification of each. It is precisely by regulating ROS, Ca^2+^, and pH in the cells, that laminarin stimulates the increase in ROS level and increases the concentration of intracellular Ca^2+^, which leads to the increase of intracellular H^+^ and acidification in the cells, then induces further downstream events of apoptosis, at last leads to the apoptosis.

### 2.3. Effect of Laminarin on Mitochondrion Permeability Transition Pore (MPTP) and Mitochondrial Membrane Potential (MMP) in LOVO Cells

**Figure 6:**
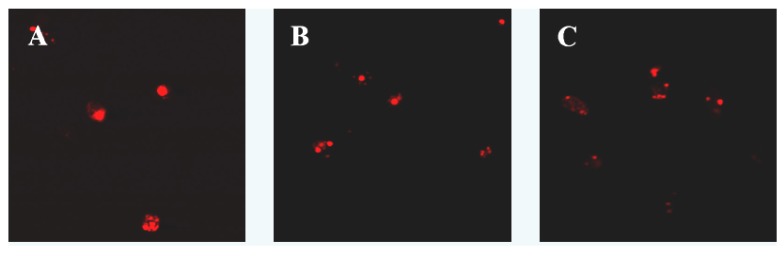

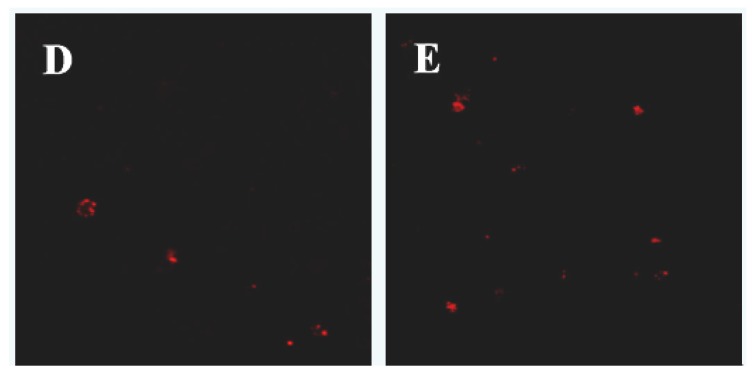
Effects of laminarin on intracellular fluorescent intensity of MPTP in LOVO. (**A**) Cells were treated as control group; (**B**) Cells were treated with 400 µg·mL^−1^ laminarin; (**C**) Cells were treated with 800 µg·mL^−1^ laminarin; (**D**) Cells were treated with 1,600 µg·mL^−1^ laminarin; (**E**) Cells were treated with 5 µg·mL^−1^ HCPT.

**Figure 7:**
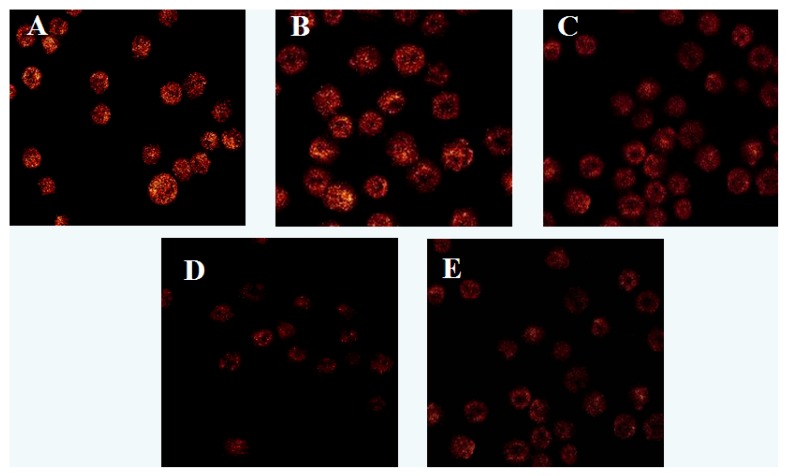
Effects of laminarin on intracellular fluorescent intensity of ΔΨm in LOVO. (**A**) Cells were treated as control group; (**B**) Cells were treated with 400 µg·mL^−1^ laminarin; (**C**) Cells were treated with 800 µg·mL^−1^ laminarin; (**D**) Cells were treated with 1,600 µg·mL^−1^ laminarin; (**E**) Cells were treated with 5 µg·mL^−1^ HCPT.

Observation with LSCM showed that after treated with different concentrations of laminarin for 24 h, the fluorescence intensity in cells weakened, which indicated the activity of MPTP increased. With the increasing drug concentration, the activity of MPTP increased accordingly, that was statistically significant compared with the control group (*p* < 0.01). After treatment with laminarin for 24 h, the fluorescence intensity in cells weakened, which indicated the level of MMP decreased. With the increasing drug concentration, the level of MMP decreased accordingly, which was statistically significant compared with the control group (*p* < 0.01) (Table 4 and Figure 6 and Figure 7).

The change in the permeability of mitochondrial membranes is the most significant event in the apoptosis process, and this permeability is controlled by MPTP, which is the key to the mitochondrial apoptosis pathway [16,17,18]. The mitochondrial membrane potential (MMP) is a result of the asymmetric distribution of protons and other ions across the inner membrane of the mitochondrion, and is necessary for sustaining the functions of the mitochondrion. It is one of the best indicators for mitochondrion permeability [19,20,21]. In our experiment, the fluorescent probes calcein/AM-cobalt and Rhodamine 123 were used for staining, and LCSM was used to observe cells. The experimental results show that, after LOVO cells were treated with laminarin for 24 h, the MPTP channels open up, MMP is lowered, and the permeability of mitochondrial membrane is increased, resulting in irreversible apoptosis in the cells.

### 2.4. Effect of Laminarin on the Expression of Cyt-C, Caspase-9 and Caspase-3 in LOVO Cells

**Figure 8:**
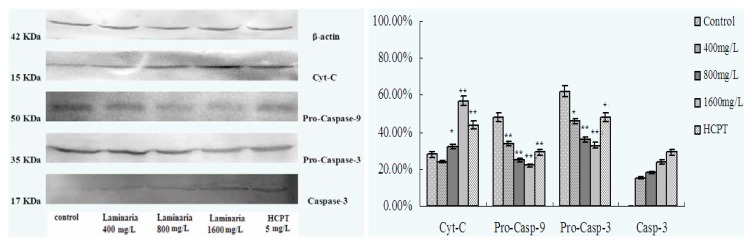
Effect of laminarin on Cyt-C, Caspase-9 and Caspase-3 expression in LOVO. (**A**) Cells were treated as control group; (**B**) Cells were treated with 400 µg·mL^−1^ laminarin; (**C**) Cells were treated with 800 µg·mL^−1^ laminarin; (**D**) Cells were treated with 1,600 µg·mL^−1^ laminarin; (**E**) Cells were treated with 5 µg·mL^−1^ HCPT. * *p <* 0.05, ** *p* < 0.01 *vs*. control group.

Analysis with Western blot showed that after treatment with different concentrations of laminarin for 24 h, the expression of Cyt-C increased, and the expression of pro-caspase-9 and pro-caspase-3 decreased gradually, while the expression of Caspase-3 increased, all in a dose-dependent manner (Figure 8). 

The release of Cyt-C is an event that occurs in the early stage of cell apoptosis [22]. Cyt-C releases into the cytoplasm through mitochondrial outer membrane permeabilization (MOMP) regulated by MPTP or members of the Bcl-2 family, and the release of Cyt-C can activate Caspase [23,24]. Caspase is a kind of proenzymes that contain no reactive site under normal conditions. In Caspase-dependent mitochondrial pathways, Cyt-C releases from the mitochondria and works with APT and Apaf-1, then converting them to polymers and promoting their combination with Caspase-9 to form apoptotic bodies [25]. It can activate Caspase-9 by hydrolyzing its proenzyme, and the activated Caspase-9 further activates Caspase-3, leading to cell apoptosis [26,27]. The experimental results show that laminarin can facilitate the release of Cyt-C from the mitochondrion into the cytoplasm, which increases the expression and activity of Caspase-9 and Caspase-3 in LOVO cells. This leads to the Caspase cascade with the formation of a Caspase-dependent pathway, thus inducing apoptosis in LOVO cells through the mitochondrial pathway.

## 3. Experimental

### 3.1. Reagents

Laminarin (Sigma-Aldrich, St. Louis, MO, USA); hydroxycamptothecin (HCPT, Harbin Shengtai Pharmaceutical Co., Ltd., Harbin, China); DMEM/F12 culture medium (Hyclone, Thermo Scientific, Waltham, MA, USA); FBS (Hangzhou Sijiqing Biological Engineering Materials Co., Ltd., Hangzhou, China); pancreatin (Gibco, Rockville, MD, USA); Rodamine123 (Sigma), Reactive Oxygen Species and pH probe (Beyotime Institute of Biotechnology, Haimen, China); Fluo-3/AM (Molecular Probes); MPTP kit (Genmed Scientifics Inc., Wilmington, DE, USA); Mouse anti-Human β-actin, Cyt-C, Caspase-9, Caspase-3 and AP AffiniPure Goat anti-Mouse (Beyotime Institute of Biotechnology).

### 3.2. Cell Culture

Human Colon Cancer Cell LOVO was provided by Center of Research and Development on Life Sciences and Environmental Sciences of Harbin University of Commerce. LOVO cells were grown in DMEM/F12 medium containing 10% heat-inactivated fetal bovine serum at 37 °C in a humidified atmosphere of 5% CO_2_.

### 3.3. Apoptosis Morphology Observation

Cells (5 × 10^4^/mL, 1 mL) were planted in 6-well plates and cultured for 24 h, then treated with different concentrations of drug for 48 h. Cells were harvested through trypsinization, and washed twice with cold PBS. The cells were centrifuged at 3,000 rpm for 5 min, then the supernatant was discarded and the pellet was resuspended in 1× binding buffer. 100 μL of the sample solution was transferred to a 5 mL culture tube, and incubated with 5 μL of FITC-conjugated annexin V and 5 μL of PI for 15 min at room temperature in the dark. 400 μL of 1× binding buffer was added to each sample tube, and the samples were analyzed by Laser Scanning Confocal Microscopy (SP2: Leica, Solms, Germany).

### 3.4. Effect of Laminarin on Reactive Oxygen Species (ROS) in LOVO Cells

Cells (5 × 10^4^/mL, 1 mL) were planted in 24-well plates and cultured for 24 h, then treated with different concentrations of drug for 24 h. Cells were digested with pancreatin and rinsed twice with PBS, loaded with DCFH-DA (5 μmol·L^−1^) for 20 min at 37 °C. The samples were rinsed once with PBS, and cells were measured using flow cytometry (EPICS XL: Beckman Coulter, Brea, CA, USA).

### 3.5. Effect of Laminarin on Calcium Ion Concertration in LOVO Cells

Cells (5 × 10^4^/mL, 1 mL) were planted in 6-well plates and cultured for 24 h, then treated with different concentrations of drug for 24 h. Cells were digested with pancreatin and rinsed twice with PBS, loaded with Fluo-3/AM (4 μg/mL) for 1 h at 37 °C. The samples were rinsed once with PBS, and cells were observed using LSCM.

### 3.6. Effect of Laminarin on pH Value in LOVO Cells

Cells (5 × 10^4^/mL, 1 mL) were planted in 24-well plates and cultured for 24 h, then treated with different concentrations of drug for 24 h. Cells were digested with pancreatin and rinsed twice with PBS, loaded with BCECF/AM (10 μmol·L^−1^) for 2 h at 37 °C. The samples were rinsed once with PBS, and cells were measured using flow cytometry.

### 3.7. Effect of Laminarin on Mitochondrion Permeability Transition Pore (MPTP) and Mitochondrial Membrane Potential (MMP) in LOVO Cells

Cells (5 × 10^4^/mL, 1 mL) were planted in 6-well plates and cultured for 24 h, then treated with different concentrations of drug for 24 h. Cells were digested with pancreatin and rinsed twice with PBS. Pro-heated Reagent A (500 μL) was added to the cells, then mixed and kept for 1 min, the supernatant was discarded by centrifugation for 10 min at 1,600 rpm. Reagent B was added to the cells and incubated for 20 min at 37 °C in the dark, and the supernatant was separated by centrifugation for 10 min at 1,600 rpm and discarded. Pro-heated Reagent A (500 μL) was added to the cells and mixed, and then cells were observed using LSCM. Cells (5 × 10^4^/mL, 1 mL) were planted in 6-well plates and cultured for 24 h, then treated with different concentrations of drug for 24 h. Cells were digested with pancreatin and rinsed twice with PBS, loaded with Rhodamine 123 (5 μg·mL^−1^) for 30 min at 37 °C. Cells were observed using LSCM.

### 3.8. Effect of Laminarin on the Expression of Cyt-C, Caspase-9 and Caspase-3 in LOVO Cells

Cells (5 × 10^4^/mL, 2 mL) were planted in 6-well plates and cultured for 24 h, then treated with different concentrations of drug for 48 h. Cytoplasm extracts were prepared with 150 μL cell lysis buffer on ice for 30 min, then the centrifuged supernatant was collected, and the protein concentration was quantified using the detergent-compatible (DC) protein assay kit. Proteins were mixed with 2× sodium dodecyl sulphate (SDS) sample buffer. A total of 40 μg of proteins were separated in a 10% (w/v) polyacrylamide gel and blotted on a nitrocellulose membrane. The blots were blocked for 2 h, and incubated with anti-Cyt-C, caspases 9 and 3, respectively, for 12 h. Subsequently, the membranes were washed in buffer and then incubated with AP Goat anti-Mouse antibody in blocking buffer. In all experiments, ponceau staining was carried out to control equal loading and the bands were visualized by electrogenerated chemiluminescence (ECL) Western blotting system. The data shown are representative of three experiments.

### 3.9. Statistical Analysis

Statistical analysis Data were expressed as mean ± standard deviation (SD). Statistical analyses were done by using the ANOVA test to compare the different groups. Probability (*p* value) of less than 0.05 was considered to be statistically significant.

## 4. Conclusions 

*Laminarin* can induce apoptosis in Human Colon Cancer LOVO Cells through a mitochondrial pathway. *Laminarin* can increase the intracellular level of ros, increase intracellular Ca^2+^, decrease intracellular pH, and induce LOVO apoptosis. *Laminarin* can open MPTP, turn on the death switch, and decrease MMP, thus inducing apoptosis by an irreversible mitochondrial pathway. *Laminarin* can adjust apoptosis-related protein expression in LOVO cells and induce apoptosis. It increases intracellular Cyt-C, activated caspase-9 and caspase-3 in a dose-dependent manner, therefore laminarin can be used as an effective drug for tumor prevention and treatment.

## Figures and Tables

**Table 1:** Effect of laminaran on ROS of LOVO cells by FCM analysis.

Groups	Concentration/(µg·mL^−1^)	MFI/($\overset{-}{x}$ ± s)
Control	0	17.1 ± 0.2
I	400	30.6 ± 0.9 **
II	800	36.0 ± 0.2 **
III	1,600	69.4 ± 0.8 **
HCPT	5	37.8 ± 0.6 **

** *p* < 0.01 *vs*. control group.

**Table 2:** Effects of laminarin on intracellular [Ca^2+^] in LOVO.

Groups	Concentration/(µg·mL^−1^)	MFI/($\overset{-}{x}$ ± s)
Control	0	24.79 ± 2.49
I	400	35.45 ± 2.82 **
II	800	46.56 ± 1.98 **
III	1,600	51.95 ± 2.39 **
HCPT	5	85.89 ± 2.21 **

** *p* < 0.01 *vs.* control group.

**Table 3:** Effect of Laminaran on pH of LOVO cells by FCM analysis.

Groups	Concentration/(µg·mL^−1^)	MFI
Control	0	20.50 ± 1.15
I	400	15.95 ± 0.76 *
II	800	13.63 ± 0.74 **
III	1,600	13.08 ± 0.58 **
HCPT	5	18.44 ± 0.45

* *p <* 0.05, ** *p* < 0.01 *vs*. control group.

**Table 4:** Effects of laminarin on activity of MPTP and ΔΨm in LOVO.

Groups	Concentration/(µg·mL^−1^)	MFI/($\overset{-}{x}$ ± s)	
		MPTP	ΔΨm
Control	0	46.40 ± 2.39	64.13 ± 4.22
I	400	38.64 ± 1.87 *	44.80 ± 1.97 **
II	800	31.30 ± 1.41 **	28.37 ± 7.85 **
III	1,600	3.62 ± 2.03 **	20.17 ± 4.06 **
HCPT	5	6.89 ± 1.38 **	27.55 ± 9.16 **

* *p* < 0.05, ** *p* < 0.01 *vs*. control group.
